# Impaired Topographical Organization of Functional Brain Networks in Parkinson’s Disease Patients With Freezing of Gait

**DOI:** 10.3389/fnagi.2020.580564

**Published:** 2020-10-21

**Authors:** Xiuhang Ruan, Yuting Li, E. Li, Fang Xie, Guoqin Zhang, Zhenhang Luo, Yuchen Du, Xinqing Jiang, Mengyan Li, Xinhua Wei

**Affiliations:** ^1^Department of Radiology, Guangzhou First People’s Hospital, School of Medicine, South China University of Technology, Guangzhou, China; ^2^Department of Radiology, Guangzhou First People’s Hospital, Guangzhou Medical University, Guangzhou, China; ^3^GYENNO Technologies Co., Ltd., Shenzhen, China; ^4^Department of Neurology, Guangzhou First People’s Hospital, School of Medicine, South China University of Technology, Guangzhou, China

**Keywords:** freezing of gait, Parkinson’s disease, graph theory, functional magnetic resonance imaging, gait disorders

## Abstract

**Objective**: This study aimed to explore alterations in the topological properties of the functional brain network in primary Parkinson’s disease (PD) patients with freezing of gait (PD-FOG).

**Methods**: Resting-state functional magnetic resonance imaging (Rs-fMRI) data were obtained in 23 PD-FOG patients, 33 PD patients without FOG (PD-nFOG), and 24 healthy control (HC) participants. The whole-brain functional connectome was constructed by thresholding the Pearson correlation matrices of 90 brain regions, and topological properties were analyzed by using graph theory approaches. The network-based statistics (NBS) method was used to determine the suprathreshold connected edges (*P* < 0.05; threshold *T* = 2.725), and statistical significance was estimated by using the non-parametric permutation method (5,000 permutations). Statistically significant topological properties were further evaluated for their relationship with clinical neurological scales.

**Results**: The topological properties of the functional brain network in PD-FOG and PD-nFOG showed no abnormalities at the global level. However, compared with HCs, PD-FOG patients showed decreased nodal local efficiency in several brain regions, including the bilateral striatum, frontoparietal areas, visual cortex, and bilateral superior temporal gyrus, increased nodal local efficiency in the left gyrus rectus. When compared with PD-nFOG patients and HCs, PD-FOG showed increased betweenness centrality in the left hippocampus. Moreover, compared to HCs, both PD-FOG and PD-nFOG patients displayed reduced network connections by using the NBS method, mainly involving the sensorimotor cortex (SM), visual network (VN), default mode network (DMN), auditory network (AN), dorsal attention network (DAN), subcortical regions, and limbic network (LIM). The local node efficiency of the right temporal pole: superior temporal gyrus in PD-FOG patients was positively correlated with the Freezing of Gait Questionnaire (FOGQ) scores.

**Conclusions**: This study demonstrates the disrupted regional topological organization in PD-FOG patients, especially associated with damage to the subcortical regions and multiple cortical regions. Our results provide insights into the dysfunctional mechanisms of the relevant networks and indicate potential neuroimaging biomarkers of PD-FOG.

## Introduction

Freezing of gait (FOG) is considered one of the most common motor symptoms in Parkinson’s disease (PD) characterized by a brief, episodic absence or unsuccessful attempts to start or turn despite the intention to walk and is often characterized as the patient having a foot “glued” to the floor. PD patients with FOG (PD-FOG) frequently fall, which can lead to disability and even lessen the quality of life (Nutt et al., 2011). Unfortunately, to date, FOG is hard to manage with medical and rehabilitation treatment or deep brain stimulation (Giladi, 2008). Several mechanisms, including motor (i.e., postural instability), cognitive (i.e., abnormal executive and visuospatial functions) as well as behavioral disorders (i.e., anxiety), have been proposed to explain FOG (Nutt et al., 2011; Factor et al., 2014). However, the pathophysiological mechanisms underlying FOG remain poorly understood.

In the past 10 years, a growing body of neuroimaging studies has been amassed that characterizes the structural and functional alterations in the brains of PD-FOG patients. Structural MRI studies have revealed gray matter loss and white matter damage in FOG patients in various cortical and subcortical regions, including frontoparietal cortical regions, several brainstem, and cerebellar nuclei, and basal ganglia (Bharti et al., 2019). Recently, the emerging notion pointed out that FOG is not only associated with damage in specific brain areas but also with brain network dysfunction. Resting-state functional magnetic resonance imaging (Rs-fMRI), which reflects baseline neuronal brain activity during rest, can be applied to detect resting-state functional connectivity (FC) and large-scale brain network organization in blood oxygen level-dependent (BOLD) signal fluctuations. Using seed-based connectivity approaches, early Rs-fMRI studies have shown greater FC in the cortical-subcortical locomotor network (Fling et al., 2014). Wang et al. (2016) found that FOG patients exhibited abnormal pedunculopontine nucleus FC mainly in the corticopontine-cerebellar pathways as well as in the visual temporal areas. Using independent component analysis (ICA), several studies have reported FC disruptions of the “executive-attention” and visual neural networks in patients with FOG (Tessitore et al., 2012; Canu et al., 2015). Together, the currently available data indicate that widespread functional disruptions in cortical and subcortical brain structures involving multiple brain networks are responsible for FOG in PD. We noticed that these studies detected coupling between symptoms and abnormal regional spontaneous neural activity or aberrant within-network intrinsic activity, rather than integrating the specificity of brain regions and connectivity of different brain regions into one framework for analysis in FOG patients. There may be new discoveries on the mechanism of FOG from the perspective of the brain’s functional network.

Recently, advances in graph-based network theory have allowed the direct noninvasive characterization of brain network topologic organization in neuropsychiatric patients (Lui et al., 2016), and it may be used to detect related network alterations of FOG. As a mature branch of mathematics, the graph theory approach describes the whole brain as a single graph composed of nodes linked by edges (Sporns et al., 2004). The representation of a “graph” and a “network” enables functional segregation and functional integration to be organically combined in a framework, allowing us to systematically investigate the neural mechanism of FOG. To date, only one study has investigated brain networks using graph analysis, which has mainly focused on two attentional networks in PD-FOG (Maidan et al., 2019). To our knowledge, whole-brain functional network properties have not been tested specifically in PD-FOG.

In the present study, we aimed to detect brain network abnormalities in PD-FOG patients and PD patients without FOG (PD-nFOG) patients using graph theory approaches and to determine their potential relationships with clinical measures. We hypothesized that the brain networks of PD patients with FOG would display disrupted functional brain topological organization compared to that of PD patients without FOG and healthy controls (HCs).

## Materials and Methods

### Participants

This study was approved by the Local Ethics Committee in Guangzhou First People’s Hospital in China, and written informed consent was obtained from all participants (or their legal guardians) before enrollment.

Patients were consecutively recruited at the neurology outpatient clinic and inpatient department of Guangzhou First People’s Hospital (Guangzhou, China) from March 2017 to November 2018. We recruited 60 right-handed patients with a diagnosis of PD that had been made by two clinical neurologists (one with 3 years and one with 20 years of experience in clinical neurology) according to the clinical diagnostic criteria of the UK Parkinson’s Disease Society (Gibb and Lees, 1988) and divided them into PD-FOG and PD-nFOG patients. Patients were classified as having FOG if they had a score ≥1 on the Freezing of Gait Questionnaire (FOGQ) item 3 (Giladi et al., 2000). FOG was observed by two experienced neurologists during a period of 10 m in which the patient walked, turned (made tight 180° turns to the right and left), and went through a narrow doorway; patients who displayed episodes of foot movement cessation were deemed to have FOG. PD-nFOG patients who were sex and gender-matched to the PD-FOG group were selected from our PD research database. The Unified Parkinson’s Disease Rating Scale (UPDRS) was used to assess mental, behavioral, and emotional (UPDRS-I), daily life (UPDRS-II), motor disability (UPDRS-III), and treatment complications (UPDRS-IV) respectively (Goetz et al., 2008). The Hoehn & Yahr stage (stages 1–5) was used to evaluate disease severity (Hoehn and Yahr, 1967), and Mini-Mental State Examination (MMSE) was used to evaluate cognition status (Folstein et al., 1983). Meanwhile, their levodopa equivalent daily dose (LEDD) was also recorded. The FOGQ and GFQ scales are evaluated under the OFF medication state, and UPDRS-III is evaluated under the ON medication state. Exclusion criteria comprised patients with deep brain stimulator use, multiple system atrophy, progressive supranuclear palsy and corticobasal degeneration with atypical parkinsonism, and excessive rest tremor.

Also, 24 right-handed HCs (12 men, 12 women) were recruited from the community by poster advertisements and assessed by a neurologist. HC participants were excluded if they had: (1) cognitive impairment (an MMSE score lower than 24); (2) any (other) major systemic, psychiatric, or neurological illnesses (i.e., depression, dementia); and (3) other causes of focal or diffuse brain damage determined *via* conventional MRI, including lacunae and extensive cerebrovascular disorders.

### MRI Data Acquisition

MRI data of all PD patients were collected during the ON medication state. Functional and structural data were acquired with a 3-Tesla system (Siemens, Erlangen, Germany) and a 16-channel phased-array head coil. Resting-state functional BOLD images were obtained by using a single-shot echo-planar imaging (EPI) sequence (33 axial slices, repetition time (TR) = 2,000 ms, echo time (TE) = 21 ms, field of view = 224 mm × 224 mm, flip angle (FA) = 78°, voxel size = 3.5 × 3 0.5 × 4.0 mm^3^, matrix = 64 × 64, slice thickness = 4.0 mm, 220 time points**)**. Anatomical images were acquired by using a 3D T1-weighted MPRAGE sequence in the sagittal orientation (TR = 1,900 ms, TE = 2.19 ms, FA = 9°, FOV = 250 mm × 250 mm, slice thickness = 1.0 mm, no gap). Axial FLAIR, T2W, T1W, and sagittal T2W were acquired to exclude subjects who had space-occupying lesions and cerebrovascular diseases.

### Preprocessing of Image Data

The processing was performed by DPARSF Advanced Edition V2.2 (Yan and Zang, 2010). These steps included: (1) the conversion of DICOM data into NIFTI data; (2) the removal of the first 10 time points; (3) slice timing correction; (4) head motion correction; (5) normalization (resampled to a voxel size of 3 × 3 × 3 mm^3^); (6) spatial smoothening (6 mm full width at half maximum); (7) filtering (0.01 < *f* < 0.08 Hz); and (8) nuisance signal regression (6 head parameters, white matter, cerebrospinal fluid (CSF) signals, and global signals). Two PD-FOG patients and two PD-nFOG patients with head motions exceeding 2 mm of translation or 2° of rotation throughout the scan were excluded from the study. Finally, we retained 23 PD-FOG patients, 33 PD-nFOG patients, and 24 HC subjects for further analyses.

### Network Construction and Analysis

The brain network was constructed (including nodes and edges) and calculated by using Gretna^1^. The whole-brain (excluding the cerebellum) was divided into 90 cortical and subcortical regions of interest (ROIs) by using automated anatomic labeling (AAL) landmarks, with each region representing a network node. The edges of the network were calculated by the Pearson correlations of the mean time-series between each pair of nodes. Therefore, this resulted in a 90 × 90 symmetric FC matrix for each participant, which was converted into a binary and undirected matrix through a predefined threshold. Specifically, we assessed the effects of thresholds over a range of sparsity (10% ≤ S ≤ 40%, at 0.05 steps), the absolute Pearson correlation between regions i and j exceeding the threshold was set to 1 or 0 otherwise.

For the network metric at each sparsity level, we calculated a series of the properties of the network (binary and undirected 90 × 90 symmetric FC matrix), including global metrics [i.e., the shortest path length (Lp), clustering coefficient (Cp), small-worldness (σ), network efficiency], and nodal property (i.e., degree centrality, betweenness centrality, nodal clustering coefficient, nodal efficiency, nodal local efficiency, and nodal shortest path). The “small world” network with a high clustering coefficient, and short shortest path length, has relatively high local and global efficiency levels in information transmission and processing. Network efficiency measures the global information transmission capability of the network.

**Figure 1:**
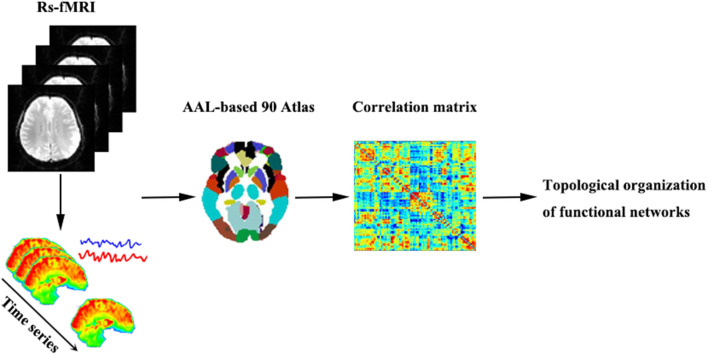
A brief flowchart showing the construction of the functional networks. Abbreviations: Rs-fMRI, resting-state functional magnetic resonance imaging; AAL, automated anatomic labeling.

Regarding regional characteristics, degree centrality reflects the importance of the node or brain region in the whole-brain network, while betweenness centrality characterizes the ability of the node to influence the entire network. Moreover, the nodal clustering coefficient measures the degree of network grouping. Node efficiency indicates the efficiency of the parallel information transmission capability of the node in the network, while nodal local efficiency measures the local information transmission capability of the network. The nodal shortest path quantifies the average distance or path efficiency between a particular node and all other nodes in the network. The processing steps of network construction are shown in Figure 1.

### Statistical Analysis

Demographics and clinical characteristics were analyzed in SPSS for Windows version 16.0^2^. Measurement data following the normal distribution were analyzed using a one-way analysis of variance (ANOVA) and a *post hoc* *t*-test. Measurement data that did not meet the normal distribution or had uneven variance were evaluated using the corresponding rank-sum test (Mann–Whitney *U* or Kruskal–Wallis H) depending on the distribution, and qualitative variables were compared using the chi-squared test. To compare parameters (global and local attributes), we adopted Bonferroni’s correction method for multiple comparisons. The network-based statistics (NBS) approach (through nonparametric permutation tests with 5,000 iterations) was used to detect altered connectivity in the whole-brain functional network between the three groups. Between-group comparisons were performed using the analysis of coANOVA, age, and gender as covariates of no interest. A two-sample *t*-test was used for *post hoc* analysis to compare the differences between each group of patients and HCs, age and gender as covariates of no interest. In particular, the post-hoc comparison of PD-FOG and PD-nFOG groups also require taking the age, gender, disease duration, and Hoehn & Yahr stage as covariates. Also, Spearman correlation analyses were performed to detect relationships between topological attributes with statistically significant differences and clinical variables with a *P-value* FDR corrected for multiple comparisons. The statistical threshold was set to *P* < 0.05 for all analyses.

## Results

### Demographic and Clinical Characteristics

The demographic and clinical data are summarized in Table 1. No significant differences were detected among the groups for gender, age or MMSE scores (*P* > 0.05). There were not significant differences in UPDRS-III (*P* = 0.820) scores between the PD-FOG and PD-nFOG groups. As expected, PD-FOG patients had a significantly higher disease duration (*P* = 0.007), H-Y (*P* = 0.018) score, UPDRS-I score (*P* = 0.046), UPDRS-II score (*P* = 0.011), UPDRS-IV score (*P* = 0.001), GFQ score (*P* = 0.001), FOGQ score (*P* = 0.001) and LEDD (*P* = 0.010) than PD-nFOG patients.

**Table 1:** Demographic and clinical characteristic of the participants.

Variables	PD-FOG (*n* = 23)	PD-nFOG (*n* = 33)	HC (*n* = 24)	*P*-values
Gender, Male/Female	11/12	18/15	12/12	0.875^d^
Age (years)	65.00 ± 6.59	63.79 ± 8.35	62.29 ± 6.03	0.189^a^
MMSE	25.63 ± 3.43	26.09 ± 4.13	27.25 ± 1.96	0.366^a^
Disease duration (years)	6.02 ± 5.27	3.27 ± 3.30	NA	0.007^b^
Hoehn & Yahr stage	2.45 ± 0.52	2.09 ± 0.46	NA	0.018^b^
UPDRS-I	2.08 ± 2.27	1.05 ± 1.51	NA	0.046^b^
UPDRS-II	10.98 ± 6.80	7.23 ± 4.12	NA	0.011^c^
UPDRS-III	28.78 ± 1.76	27.86 ± 1.32	NA	0.820^c^
UPDRS-IV	2.87 ± 2.94	0.77 ± 1.40	NA	0.001^b^
GFQ	16.22 ± 1.27	3.4 ± 2.79	NA	0.001^b^
FOGQ	9.59 ± 6.41	1.60 ± 1.64	NA	0.001^b^
LEDD	494.51 ± 309.35	316.63 ± 188.76	NA	0.010^c^

*Data were presented as means ± standard deviations. Abbreviations: PD-FOG, Parkinson’s disease with freezing of gait; PD-nFOG, Parkinson’s disease without freezing of gait; MMSE, Mini-Mental State Examination; UPDRS, Unified Parkinson’s Disease Rating Scale; GFQ, Gait and Falls Questionnaire; FOGQ, Freezing of Gait Questionnaire; NA, not applicable. ^a^*P*-values for comparisons between the three groups obtained using Kruskal-Wallis H test; ^b^*P*-values for differences between PD-FOG and PD-nFOG participants obtained using Mann-Whitney *U* test; ^c^*P*-values for comparisons between PD-FOG and PD-nFOG participants obtained two-sample *t*-test; ^d^*P*-values obtained using χ^2^ test between the three groups*.

### Global Topological Organization Analysis

In the defined threshold range, both groups of PD patients and HCs demonstrated small-world topological properties (σ > 1). However, no significant differences in the small-worldness, clustering coefficient, normalized clustering coefficient, shortest path length, or normalized shortest path length were observed among the groups by ANOVA (*P* > 0.05). Also, network efficiency among the groups was not significantly different by ANOVA (*P* > 0.05).

### Regional Topologic Organization Analysis

**Figure 2:**
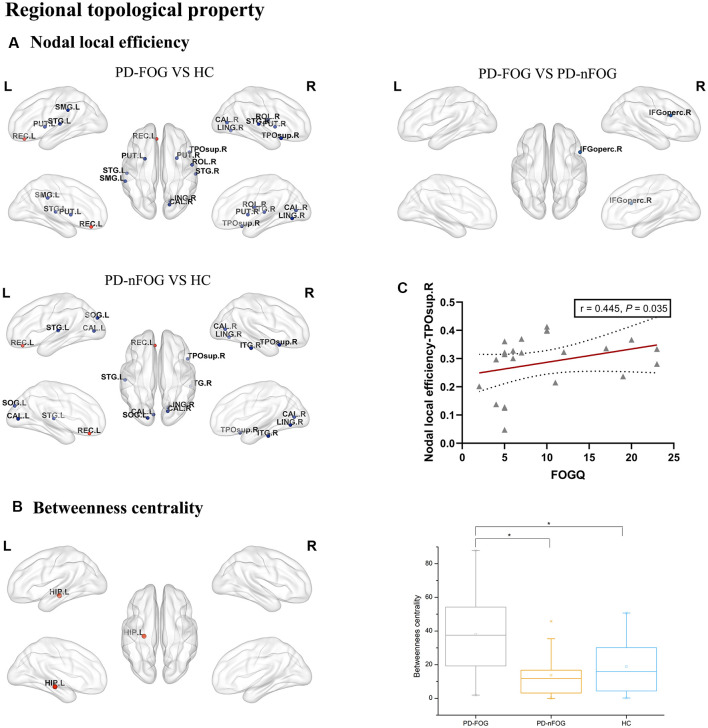
Brain regions showing abnormal regional topologic properties in PD-FOG patients relative to PD-nFOG patients and HCs. The cold color representing the decreased regional topologic properties, and the warm color representing the increased regional topologic properties. **(A)** Brain regions showing abnormal nodal local efficiency in PD-FOG patients relative to PD-nFOG patients and HCs. **(B)** Brain regions showing increased betweenness centrality in the left hippocampus (HIP.L) in PD-FOG patients compared with PD-nFOG patients and HCs. **(C)** Plots showing the correlation between nodal local efficiency in the right temporal pole: superior temporal gyrus and FOGQ scores in PD-FOG patients. *Means a significant statistical difference between the two groups. Network visualization was obtained with BrainNet viewer (http://www.nitrc.org/projects/bnv/) software packages. Abbreviations: PD-FOG, Parkinson’s disease patients with freezing of gait; PD-nFOG, Parkinson’s disease patients without freezing of gait; HC, healthy control; FOGQ, Freezing of Gait Questionnaire; ROL, Rolandic operculum; REC, Gyrus rectus; CAL, Calcarine fissure and surrounding cortex; LING, Lingual gyrus, SMG, Supramarginal gyrus; PUT, Putamen; STG, superior temporal gyrus; TPOsup, Temporal pole: superior temporal gyrus; IFGoperc, Inferior frontal gyrus, opercular part; ITG, inferior temporal gyrus; L, Left; R, Right.

The ANOVA test revealed that the nodal local efficiency of the PD-FOG, PD-nFOG and HC groups were significantly different. *Post hoc* comparisons between groups indicated decreased nodal local efficiency in right rolandic operculum, right calcarine fissure and surrounding cortex, left lingual gyrus, left supramarginal gyrus, left putamen, right putamen, left superior temporal gyrus, right superior temporal gyrus, right temporal pole: superior temporal gyrus, increased nodal local efficiency in the left gyrus rectus in PD-FOG patients compared with HCs (*P* = 0.026, *P* = 0.045, *P* = 0.022, *P* = 0.040, *P* = 0.0005, *P* = 0.011, *P* = 0.003, *P* = 0.015, *P* = 0.036, *P* = 0.020, respectively, Bonferroni’s corrected; Figure 2A). Compared with HCs, the PD-nFOG group exhibited decreased nodal local efficiency in left calcarine fissure and surrounding cortex, right calcarine fissure and surrounding cortex, left lingual gyrus, right lingual gyrus, left superior temporal gyrus, right temporal pole: superior temporal gyrus, right inferior temporal gyrus, increased nodal local efficiency in the left gyrus rectus (*P* = 0.0004, *P* = 0.011, *P* = 0.027, *P* = 0.017, *P* = 0.031, *P* = 0.040, *P* = 0.030, *P* = 0.023, respectively, Bonferroni’s corrected; Figure 2A). Compared with PD-nFOG, the PD-FOG group exhibited decreased nodal local efficiency in right inferior frontal gyrus, opercular part (*P* = 0.040, Bonferroni’s corrected; Figure 2A). Obviously, the nodal local efficiency of PD-FOG is more serious than that of PD-nFOG.

Also, significant group effects on betweenness centrality were observed among the PD-FOG, PD-nFOG, and HC groups. *Post hoc* comparisons between groups indicated increased betweenness centrality in the left hippocampus in PD-FOG patients compared with PD-nFOG patients and HCs (*P* = 0.000135, *P* = 0.000328, respectively, Bonferroni’s corrected; Figure 2B), while there was no statistically significant difference between PD-nFOG patients and HCs.

Also, there were no statistically significant differences in the degree centrality, nodal clustering coefficient, nodal efficiency, or nodal shortest path among the three groups by ANOVA (*P* > 0.05).

### PD-FOG and PD-nFOG Related Alterations in Functional Connectivity Characteristics

**Figure 3:**
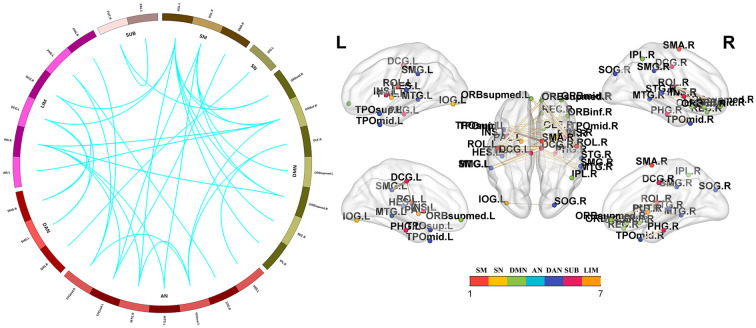
Functional connectivity network showing decreased functional connections in PD-FOG patients. Compared with the HC group, the PD-FOG group showed a decrease in multiple nodes and connections, which included 29 nodes and 35 connections, mainly involving the sensorimotor cortex (SM), visual network (VN), default mode network (DMN), auditory network (AN) dorsal attention network (DAN), subcortical regions (SUBs) and limbic network (LIM). Network visualization was obtained with BrainNet viewer (http://www.nitrc.org/projects/bnv/) software packages. Abbreviations: PD-nFOG, Parkinson’s disease patients without freezing of gait; HC, healthy control.

**Figure 4:**
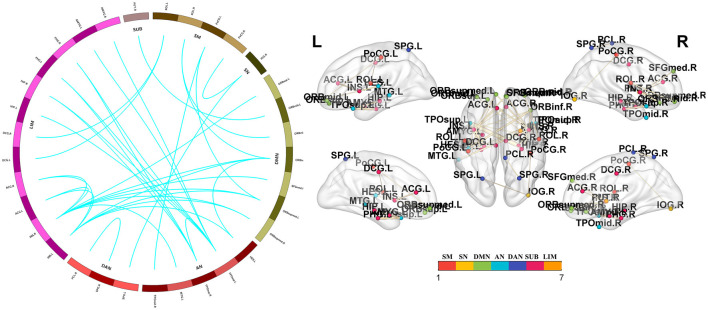
Functional connectivity reductions in PD-nFOG patients. Compared with HCs, PD-nFOG patients show reduced functional connections in multiple brain networks, including 33 nodes and 35 connections (*T* > 4), which mainly involved the sensorimotor cortex (SM), visual network (VN), default mode network (DMN), auditory network (AN) dorsal attention network (DAN), subcortical regions (SUBs) and limbic network (LIM). Network visualization was obtained with BrainNet viewer (http://www.nitrc.org/projects/bnv/) software packages. Abbreviations: PD-nFOG, Parkinson’s disease patients without freezing of gait; HC, healthy control.

Significant group differences in whole-brain FC were noted among the PD-FOG, PD-nFOG, and HC groups (*P* = 0.024, NBS-corrected). *Post hoc* comparisons between groups and FDR correction results showed that Compared with HCs, PD-FOG patients showed decreased functional connections in multiple nodes, including 29 nodes and 35 connections (*P* < 0.05, FDR-corrected), mainly involving the sensorimotor cortex (SM), visual network (VN), default mode network (DMN), auditory network (AN), dorsal attention network (DAN), subcortical regions and limbic network (LIM; Figure 3 and Supplementary Table 1). Compared with HCs, PD-nFOG patients showed reduced functional connections in multiple brain networks, including 47 nodes and 71 connections (*P* < 0.05, FDR-corrected), when T was > 4, functional connections were still decreased in 33 nodes and 35 connections, mainly involving the SM, VN, DMN, AN, DAN, subcortical regions and LIM (Figure 4 and Supplementary Table 2). No significant difference in whole-brain FC was observed between PD-FOG and PD-nFOG patients.

### Correlation Analysis

In PD-FOG patients, the nodal local efficiency in the right temporal pole: superior temporal gyrus was positively correlated with FOGQ (*P* = 0.035, *r* = 0.445, FDR-corrected; Figure 2C).

## Discussion

Using Rs-fMRI and graph theory network analysis, we found disrupted functional network topographical organization in PD-FOG patients. Our main findings are as follows: (1) regarding the global topological organization, the PD-FOG, PD-nFOG, and HC groups all exhibited small-world attributes. (2) Regarding regional topological organization, PD-FOG patients demonstrated decreased nodal local efficiency in multiple brain regions, including the bilateral striatum, frontoparietal areas, visual cortex, and bilateral superior temporal gyrus, increased nodal local efficiency in the left gyrus, and increased betweenness centrality in the left hippocampus rectus. (3) Based on the NBS approach, decreased connections were observed not only in PD-FOG but also in PD-nFOG patients, mainly found in the SM, VN, DMN, AN, DAN, subcortical regions, and LIM. Also, the nodal local efficiency of the right temporal pole: superior temporal gyrus was positively correlated with FOGQ in PD-FOG patients. These findings have promoted our understanding of the underlying neural mechanisms of FOG from a network perspective.

Under any network sparsity, no group effect was found in the global topological organization (including small-worldness, network efficiency). In the framework of graph theory, small-worldness properties reflect the best balance between local specialization (indexed by a high clustering coefficient) and global integration (indexed by low characteristic path length; Sporns et al., 2005), and its damage mainly exists in mental disorders caused by physical diseases, organic mental disorders and some more serious mental diseases, such as cardiovascular and cerebrovascular diseases, epilepsy, schizophrenia, Alzheimer’s disease and so on. Previous studies have shown that the brain networks of PD patients and HC participants showed small-worldness (Ma et al., 2017; Suo et al., 2017), which is mostly consistent with the results of this study. Also, our study did not find a significant change in the global topological organization of the PD-FOG network. It is inconsistent with previous studies, but previous studies only focused on changes in the network attributes of the ventral and ventral attention networks and only found that the global efficiency of the DAN was reduced (Maidan et al., 2019), suggesting that the global topology of the PD-FOG has not changed significantly. It further shows that the difference between PD-FOG and PD-NFOG mainly lies in the changes in the regional topologic organization of the brain functional network.

In addition to altered global topological properties, compared with HCs, PD-FOG patients showed decreased nodal local efficiency in several brain regions, including the bilateral striatum, frontoparietal areas, visual cortex, and bilateral superior temporal gyrus, increased nodal local efficiency in the left gyrus rectus. Nigrostriatal losses of PD-FOG patients have been reported in previous studies (Perez-Lloret et al., 2014), and damage to the VN and frontoparietal network have also been reported (Fasano et al., 2015). The frontal-parietal network participates in executive attention functions. Superior temporal gyrus is related to emotional perception in facial stimulation and plays a role in regulating attention deviation (Belzung et al., 2015). Although these areas may not be directly related to the onset of PD-FOG, their functional impairment may be one of the potential pathogenesis of PD-FOG. Meanwhile, extra-nigral pathologies may damage the information flow of the cerebellum, thalamus, and cortex (Sarter et al., 2014), thus weakening the visual and executive attention information entering the striatal motor network, which may lead to the inability to detect relevant motor cues in the basal ganglia. The interruption of this information flow will further aggravate impaired motor selection and sequencing, thus increasing the risk of FOG (Weiss et al., 2020). Moreover, there were significant positive correlations between the nodal local efficiency of the right temporal pole: superior temporal gyrus and FOGQ score in PD-FOG patients. It shows that as the FOG symptoms aggravate, the efficiency of the local nodes of the right temporal pole becomes higher, suggesting that the local topological attributes are helpful to detect the neuromodulation related to disease progression in the right temporal pole, and therefore serve as potential biomarkers for disease progression. Also, the frontal lobe is mainly related to executive function and working memory. Some studies have suggested that the decrease of frontal lobe activation is related to the impairment of executive function in PD-FOG patients (Zhou et al., 2018). In this study, the increased neuron activity in some areas of the frontal lobe in the PD-FOG group may be a compensatory trend for potential cognitive decline, but due to this study did not conduct a detailed assessment of cognition, and further follow-up studies are needed. Compared with HCs, PD-nFOG patients showed decreased nodal local efficiency in the bilateral visual cortex and bilateral superior temporal gyrus, increased nodal local efficiency in the left gyrus rectus, the main difference with PD-FOG is that the nodal local efficiency damage of PD-nFOG is not so serious. Compared with PD-nFOG, the PD-FOG group exhibited decreased nodal local efficiency in the right inferior frontal gyrus, opercular part. A resting-state study suggested that decreased ReHo in the inferior frontal gyrus may cause damage to the executive function of PD-FOG patients (Liu et al., 2019). Therefore, the reduction of nodal local efficiency in inferior frontal gyrus is an important factor causing FOG, and it is also one of the important distinctions between PD-FOG and PD-NFOG.

Other than the changes in nodal local efficiency, PD-FOG showed increased betweenness centrality in the left hippocampus when compared with PD-nFOG patients and HCs. Hippocampus is related to the task performance of studying set-shifting and working memory. Therefore, changes in betweenness centrality in areas related to cognitive performance may affect effective information transmission in the network and may explain the occurrence of FOG. Research by Brown et al. found that the participation coefficient of the hippocampus was decreased, indicating that changes in structural topological properties of hippocampal are involved in the pathogenesis of FOG (Hall et al., 2018). However, in the FOG group, the left hippocampus showed a higher betweenness centrality, differences in research content should be considered to explain the differences between the results.

In addition to investigating topological properties, we also compared brain network FC among the three groups. NBS analyses showed that multiple brain network connections were decreased in PD-FOG and PD-nFOG patients, mainly involved the SM, VN, DMN, AN, DAN, subcortical regions, and LIM. It is well known that the early signs of PD are a decrease in dopamine input in the cortex and subcortical structure (Rodriguez-Oroz et al., 2009). Previous results agree with this finding and showed that PD patients have abnormal white matter integrity and specific functional network changes in the SM (Sharman et al., 2013) and subcortical regions (Owens-Walton et al., 2018). Furthermore, Visual function is also the main complex sensory area affected by PD. The nodal centrality and network connection capability of the temporal-occipital region of PD patients are reduced, suggesting that these regions are related to impaired bottom-up visual processing (Luo et al., 2015; Suo et al., 2017). Sensorimotor and visual areas are important for motor learning and motor control, especially the visual-sensorimotor interaction, which is lacking in PD patients (Inzelberg et al., 2008). Moreover, the DMN is an advanced cognitive network and is related to the ability to process self-references (Gusnard and Raichle, 2001; Mak et al., 2017). The DAN also plays an important role in cognitive processing. Previous studies have also confirmed that PD patients with cognitive impairment have altered FC in the default network and DAN (Amboni et al., 2015; Baggio et al., 2015; Lucas-Jimenez et al., 2016). Although the enrolled most of the patients had no clinical manifestations of cognitive impairment, they showed abnormalities in cognitive-related brain regions, which may indicate that early neural function changes in the DMN and DAN may predict cognitive decline. Furthermore, PD-FOG and PD-NFOG patients also showed decreased connections in the AN. It has been reported that external sensory input, such as rhythmic auditory stimuli and listening to instructions while walking on a striped floor, can improve the motor symptoms of PD (Hausdorff et al., 2007; Lee et al., 2012). This finding partially suggests that external auditory stimulation can improve the motor symptoms of PD. Our study also found that PD patients with or without FOG have reduced connectivity in the LIM. Some clinical studies have shown that PD patients had significant anosmia 4–5 years before the onset of motor symptoms. Using rs-fMRI studies, Su et al. (2015) found that PD with olfactory dysfunction was related to the reduction of FC in some non-traditional olfactory areas in the limbic/paralimbic cortices. Therefore, it suggests that the reduced connectivity in the LIM may be related to PD itself, but the relationship with PD-related gait abnormalities is not clear. Most of the research results of the Parkinson subgroup in these two groups were similar to previous research results, suggesting that the mechanism of PD is related to the sensorimotor network, VN, DMN, AN, DAN, subcortical regions, and LIM dysfunction. The results of this study provide a theoretical basis for the establishment of clinical targeted treatment measures.

## Limitations

The current study has several limitations. First, we used an AAL atlas to divide the entire brain into 90 brain regions, without the cerebellum, but brain networks derived from different brain atlases show different topological organizations (Wang et al., 2009). Second, functional MRI data were acquired and part of the clinical evaluation scale of all PD patients was performed during an ON medication state; further research is needed to quantify the potential difference between ON and OFF states to assess the effect of the medication status on the topological characteristics of brain networks. Third, the sample size of this study is relatively small, larger samples and longitudinal follow-ups are still needed for further research.

## Conclusions

In summary, we used graph theory approaches to study changes in brain functional network topographic organization in PD-FOG patients. The present study demonstrated that PD-FOG patients exhibited reduced nodal local efficiency in the bilateral striatum, frontoparietal areas, visual cortex, and bilateral superior temporal gyrus, increased nodal local efficiency in the left gyrus, and increased betweenness centrality in left hippocampus rectus, the nodal local efficiency of the right temporal pole: superior temporal gyrus was positively correlated with FOGQ score. Moreover, PD patients showed decreased network connections in the SM, VN, DMN, AN, DAN, subcortical regions, and LIM. Taken together, these findings may provide ideas for neurobiological PD-FOG research and help to pinpoint imaging biomarkers for making an early diagnosis, identifying, treating, and determining the prognosis of PD-FOG patients.

## Data Availability Statement

The raw data supporting the conclusions of this article will be made available by the authors, without undue reservation.

## Ethics Statement

The studies involving human participants were reviewed and approved by the Local Ethics Committee in Guangzhou First People’s Hospital in China. The patients/participants provided their written informed consent to participate in this study.

## Author Contributions

XJ, ML, and XW were responsible for the conception, study design, review, and critique. YL, GZ, FX, ZL, and YD contributed to the collection and analysis of data. XR and EL were responsible for the drafting of the manuscript and figures.

## Conflict of Interest

ZL was employed by the company GYENNO Technologies Company Limited.

The remaining authors declare that the research was conducted in the absence of any commercial or financial relationships that could be construed as a potential conflict of interest.

## Footnotes

^1^http://www.nitrc.org/projects/gretna/

^2^http://www.spss.com
