# Supplementary material for: Impaired Topographical Organization of Functional Brain Networks in Parkinson’s Disease Patients With Freezing of Gait
Source: Front Aging Neurosci. 2020 Oct 21;12:580564. doi: 10.3389/fnagi.2020.580564 (PMC7609969; doi:10.3389/fnagi.2020.580564)
Supplement: Supplementary file 1 [file Table_1.PDF]

Table S1. Decreased functional connections in the PD-FOG patients compared to health controls

Num. of region 1	Brain region	Num. of region 2	Brain region	*T*-value
**10**	Middle frontal gyrus, orbital part. R	**17**	Rolandic operculum. L	3.929
**10**	Middle frontal gyrus, orbital part. R	**74**	Putamen. R	4.018
**16**	Inferior frontal gyrus, orbital part. R	**17**	Rolandic operculum. L	4.343
**16**	Inferior frontal gyrus, orbital part. R	**18**	Rolandic operculum. R	3.887
**16**	Inferior frontal gyrus, orbital part. R	**29**	Insula. L	3.832
**16**	Inferior frontal gyrus, orbital part. R	**30**	Insula. R	3.839
**16**	Inferior frontal gyrus, orbital part. R	**83**	Temporal pole: superior temporal gyrus. L	4.321
**17**	Rolandic operculum. L	**26**	Superior frontal gyrus, medial orbital.R	4.356
**17**	Rolandic operculum. L	**28**	Gyrus rectus. R	4.149
**17**	Rolandic operculum. L	**33**	Median cingulate and paracingulate gyri. L	4.006
**17**	Rolandic operculum. L	**34**	Median cingulate and paracingulate gyri. R	4.767
**17**	Rolandic operculum. L	**62**	Inferior parietal, but supramarginal and angular gyri. R	3.878
**17**	Rolandic operculum. L	**86**	Middle temporal gyrus. R	3.891
**18**	Rolandic operculum. R	**25**	Superior frontal gyrus, medial orbital. L	4.262
**18**	Rolandic operculum. R	**26**	Superior frontal gyrus, medial orbital. R	4.334
**18**	Rolandic operculum. R	**85**	Middle temporal gyrus. L	3.954
**18**	Rolandic operculum. R	**87**	Temporal pole: middle temporal gyrus. L	3.986
**20**	Supplementary motor area. R	**87**	Temporal pole: middle temporal gyrus. L	4.268
**22**	Olfactory cortex. R	**79**	Heschl gyrus. L	3.881
**22**	Olfactory cortex. R	**83**	Temporal pole: superior temporal gyrus. L	4.022
**26**	Superior frontal gyrus, medial orbital.R	**30**	Insula. R	4.027
**30**	Insula. R	**85**	Middle temporal gyrus. L	4.563
**30**	Insula. R	**86**	Middle temporal gyrus. R	3.975
**30**	Insula. R	**87**	Temporal pole: middle temporal gyrus. L	4.404
**33**	Median cingulate and paracingulate gyri. L	**63**	Supramarginal gyrus. L	4.401

**33**	Median cingulate and paracingulate gyri. L	**64**	Supramarginal gyrus. R	4.023
**34**	Median cingulate and paracingulate gyri. R	**63**	Supramarginal gyrus. L	4.634
**34**	Median cingulate and paracingulate gyri. R	**64**	Supramarginal gyrus. R	4.036
**39**	Parahippocampal gyrus. L	**82**	Superior temporal gyrus. R	3.901
**39**	Parahippocampal gyrus. L	**88**	Temporal pole: middle temporal gyrus. R	5.120
**40**	Parahippocampal gyrus. R	**88**	Temporal pole: middle temporal gyrus. R	4.247
**50**	Superior occipital gyrus. R	**53**	Inferior occipital gyrus. L	3.924
**74**	Putamen. R	**75**	Pallidum. L	3.946
**83**	Temporal pole: superior temporal gyrus. L	**86**	Middle temporal gyrus. R	4.330
**83**	Temporal pole: superior temporal gyrus. L	**88**	Temporal pole: middle temporal gyrus. R	3.850

The region pairs showing decreased functional connections in PD-FOG patients. These connections formed a connected network identified by a network-based statistic approach ($p < 0.05$, false discovery rate corrected). See Fig.3 for the matrix presentation of these connections. Abbreviations: PD-FOG, Parkinson’s disease patients with freezing of gait; L, left; R, right.

Table S2. Decreased functional connections in the PD-nFOG patients compared to health controls

**Num. of region 1**	**Brain region**	**Num. of region 2**	**Brain region**	***T*-value**
**5**	Superior frontal gyrus, orbital part. L	**30**	Insula. R	4.142
**9**	Middle frontal gyrus, orbital part. L	**74**	Putamen. R	4.151
**10**	Middle frontal gyrus, orbital part. R	**18**	Rolandic operculum. R	4.326
**16**	Inferior frontal gyrus, orbital part. R	**18**	Rolandic operculum. R	4.088
**16**	Inferior frontal gyrus, orbital part. R	**29**	Insula. L	4.323
**16**	Inferior frontal gyrus, orbital part. R	**30**	Insula. R	4.690
**16**	Inferior frontal gyrus, orbital part. R	**79**	Heschl gyrus. L	4.890
**17**	Rolandic operculum. L	**33**	Median cingulate and paracingulate gyri. L	4.173
**17**	Rolandic operculum. L	**34**	Median cingulate and paracingulate gyri. R	4.007
**24**	Superior frontal gyrus, medial. R	**30**	Insula. R	4.337
**25**	Superior frontal gyrus, medial orbital. L	**29**	Insula. L	4.320
**25**	Superior frontal gyrus, medial orbital. L	**30**	Insula. R	4.273
**25**	Superior frontal gyrus, medial orbital. L	**57**	Postcentral gyrus. L	4.202
**26**	Superior frontal gyrus, medial orbital. R	**29**	Insula. L	4.389
**26**	Superior frontal gyrus, medial orbital. R	**30**	Insula. R	4.428
**29**	Insula. L	**31**	Anterior cingulate and paracingulate gyri. L	4.614
**29**	Insula. L	**32**	Anterior cingulate and paracingulate gyri. R	4.280
**29**	Insula. L	**79**	Heschl gyrus. L	4.476
**30**	Insula. R	**31**	Anterior cingulate and paracingulate gyri. L	4.497
**30**	Insula. R	**32**	Anterior cingulate and paracingulate gyri. R	4.277
**30**	Insula. R	**85**	Middle temporal gyrus.L	4.700
**37**	Hippocampus. L	**83**	Temporal pole: superior temporal gyrus. L	4.536
**38**	Hippocampus. R	**83**	Temporal pole: superior temporal gyrus. L	4.272
**38**	Hippocampus. R	**84**	Temporal pole: superior temporal gyrus. R	4.474
**39**	Parahippocampal gyrus. L	**83**	Temporal pole: superior temporal gyrus. L	5.361
**39**	Parahippocampal gyrus. L	**84**	Temporal pole: superior temporal gyrus. R	4.418
**40**	Parahippocampal gyrus. R	**83**	Temporal pole: superior temporal gyrus. L	6.370

**40**	Parahippocampal gyrus. R	**84**	Temporal pole: superior temporal gyrus. R	4.842
**41**	Amygdala. L	**83**	Temporal pole: superior temporal gyrus. L	4.634
**42**	Amygdala. R	**83**	Temporal pole: superior temporal gyrus. L	4.484
**54**	Inferior occipital gyrus. R	**58**	Postcentral gyrus. R	4.212
**54**	Inferior occipital gyrus. R	**59**	Superior parietal gyrus. L	4.228
**57**	Postcentral gyrus. L	**88**	Temporal pole: middle temporal gyrus. R	4.227
**60**	Superior parietal gyrus. R	**70**	Paracentral lobule. R	4.308
**83**	Temporal pole: superior temporal gyrus. L	**84**	Temporal pole: superior temporal gyrus.R	4.029

The region pairs showing decreased functional connections in PD-nFOG patients. These connections formed a connected network identified by a network-based statistic approach ($p < 0.05$, false discovery rate corrected). The matrix representation of these connections is shown in figure. 4. Abbreviations: PD-nFOG, Parkinson’s disease patients without freezing of gait; L, left; R, right.
